# Prolonged static stretching increases the magnitude and decreases the complexity of knee extensor muscle force fluctuations

**DOI:** 10.1371/journal.pone.0288167

**Published:** 2023-07-21

**Authors:** Jamie Pethick, Jason Moran, David G. Behm

**Affiliations:** 1 School of Sport, Rehabilitation and Exercise Sciences, University of Essex, Colchester, Essex, United Kingdom; 2 School of Human Kinetics and Recreation, Memorial University of Newfoundland, St. John’s, Newfoundland and Labrador, Canada; Università degli Studi di Milano: Universita degli Studi di Milano, ITALY

## Abstract

Static stretching decreases maximal muscle force generation in a dose-response manner, but its effects on the generation of task-relevant and precise levels of submaximal force, i.e. force control, is unclear. We investigated the effect of acute static stretching on knee extensor force control, quantified according to both the magnitude and complexity of force fluctuations. Twelve healthy participants performed a series of isometric knee extensor maximal voluntary contractions (MVCs) and targeted intermittent submaximal contractions at 25, 50 and 75% MVC (3 x 6 seconds contraction separated by 4 seconds rest, with 60 seconds rest between each intensity) prior to, and immediately after, one of four continuous static stretch conditions: 1) no stretch; 2) 30-second stretch; 3) 60-second stretch; 4) 120-second stretch. The magnitude of force fluctuations was quantified using the standard deviation (SD) and coefficient of variation (CV), while the complexity of fluctuations was quantified using approximate entropy (ApEn) and detrended fluctuation analysis (DFA) α. These measures were calculated using the steadiest 5 seconds of the targeted submaximal contractions at each intensity (i.e., that with the lowest SD). Significant decreases in MVC were evident following the 30, 60 and 120-second stretch conditions (all *P* < 0.001), with a significant correlation observed between stretch duration and the magnitude of decrease in MVC (*r* = –0.58, *P* < 0.001). The 120-second stretch resulted in significant increases in SD at 50% MVC (*P* = 0.007) and CV at 50% (*P* = 0.009) and 75% MVC (*P* = 0.005), and a significant decrease in ApEn at 75% MVC (*P* < 0.001). These results indicate that the negative effects of prolonged static stretching extend beyond maximal force generation tasks to those involving generation of precise levels of force during moderate- to high-intensity submaximal contractions.

## Introduction

The necessity of a pre-activity warm-up for combat, work or sport has been evident for millennia [1]. Typically, this would comprise a submaximal aerobic component, a bout of stretching, and a period of specific skill rehearsal (i.e., dynamic movements similar to those performed in the sport/event) [2]. With regard to stretching, four main types are recognised: static, dynamic, ballistic, and proprioceptive neuromuscular facilitation [2]; though for many years static stretching (SS) has been the predominant form incorporated into warm-ups [3]. SS involves lengthening a muscle either until a stretch sensation is experienced [4] or the point of discomfort is reached [5] and holding the muscle in that position for 15 to 60 seconds [6]. The traditional goals of SS are to increase range of motion, decrease musculotendinous injury incidence and improve exercise performance [1, 3]. Whilst there is strong evidence for improving range of motion [7], accumulating evidence suggests that prolonged (>60 seconds per muscle group) SS without a proper warm-up impairs, rather than enhances, subsequent exercise performance [8].

Research has demonstrated that SS prior to exercise impairs maximal muscular performance, measured by decreases in one repetition maximum [9] and maximal voluntary isometric contraction (MVC) [10, 11]. Moreover, there appears to be a dose-response relationship between stretch duration and the magnitude of maximal muscular performance deficit [12, 13]. Stretch durations of <60 seconds generally have trivial effects on performance, whereas stretch durations of ≥60 seconds result in significant, practically relevant deficits in maximal force [8, 13]. Accordingly, it has been recommended to avoid performing prolonged SS prior to tasks necessitating maximal force generation [14]. The ability to generate maximal force is not, however, the sole determinant of exercise performance. The ability to control muscle force, i.e., to generate task-relevant and precise levels of force [15], is also important in determining performance, and has yet to be investigated in the context of SS.

Muscle force output is characterised by constant fluctuations, indicating that control of force is not perfectly accurate. Long thought of as unwanted noise, these fluctuations are now regarded as a rich source of information about the neural mechanisms underlying motor behaviour [16]. Traditionally, such fluctuations were quantified according to their magnitude, using the standard deviation (SD) and coefficient of variation (CV), which provide an index of the degree of deviation from a fixed point within a time-series and assume that fluctuations are random and independent [17]. Advances in analytical techniques have led to the recognition that fluctuations in muscle force are neither random nor independent, but rather possess a statistically irregular temporal structure (i.e., complexity) [17]. Complexity measures quantify the temporal irregularity (e.g., approximate entropy; ApEn) [18] and long-range fractal correlations (e.g., detrended fluctuation analysis α; DFA) [19] exhibited by muscle force; properties that magnitude-based measures cannot quantify [20]. Consequently, it has been recommended that magnitude- and complexity-based measures be used in conjunction to provide a more complete understanding of muscle force control [15].

Alterations in muscle force control can have significant impact on exercise performance. The development of neuromuscular fatigue, for example, is characterised by an increase in the magnitude and decrease in the complexity [21] of force fluctuations, indicative of decreased force steadiness and decreased adaptability [22], respectively. Such changes have been speculated to affect co-ordination, increase the risk of failing motor tasks, and play a role in task failure during neuromuscular fatigue [15], all of which could result in poorer performance of skilled movements in an exercise performance context.

Changes in the ensemble behaviour of the motor unit pool (i.e., motor unit recruitment, discharge rates) are thought to be responsible for alterations in force control [23]. Several studies have observed that the SS-induced deficits in maximal force production are associated with alterations in motor unit activation [10] and discharge rates [24, 25], indicative of an increase in the neural drive required to achieve a given target force [26]. This suggests that SS has the potential to impact muscle force control. Moreover, it has been argued that these changes in motor unit behaviour are unaffected by SS durations of <60 seconds [2], suggesting a potential dose-response relationship between SS and muscle force control. The purpose of the present study was, therefore, to investigate the effect of acute SS on muscle force control, quantified according to both the magnitude (i.e., SD, CV) and complexity (i.e., ApEn, DFA α) of force fluctuations. It was hypothesised that prolonged (i.e., >60 seconds) SS would lead to an increase in the magnitude and decrease in the complexity of knee extensor force fluctuations, responses indicative of a loss of force control.

## Methods

### Participants

Statistical power was calculated based on previous studies investigating MVC and SS [11, 27]. These calculations indicated that a sample size of between 5 and 14 participants would be necessary to attain adequate statistical power (*P* < 0.05, power = 0.8; G*Power 3.1, University of Düsseldorf, Germany). Twelve healthy male participants (mean ± SD: age 23.8 ± 5.3 years; height 1.77 ± 0.05 m; body mass 76.8 ± 6.2 kg) provided written informed consent to participant in the study, which had been approved by the ethics committee of the University of Essex (ref. ETH2021-1981), and which adhered to the Declaration of Helsinki. All participants were either recreationally active (taking part in sports including football, rugby, tennis, and basketball) or were engaged in resistance training, taking part in these activities two-three times per week. Participants were recruited to the study only after ethics approval had been granted. Exclusion criteria were any lower-limb injuries in the past three months. Participants were instructed to arrive at the laboratory in a rested state (i.e., no strenuous exercise in the preceding 24 hours) and to have consumed neither any food nor caffeinated beverages in the 3 hours prior to arrival. Participants visited the laboratory at the same time of day (± 2 hours).

### Experimental design

Participants visited the laboratory on five occasions, with a minimum of 48 hours between each visit. During their first visit, participants were familiarised with all testing equipment/procedures, and the settings for the dynamometer were recorded. During the next four visits, participants performed a series of targeted intermittent isometric knee extension contractions prior to, immediately after, and ten minutes after, one of four knee extensor SS conditions (presented in a randomised order): 1) no stretch (i.e., control); 2) 30-second stretch; 3) 60-second stretch; and 4) 120-second stretch. During the contractions, muscle force output was sampled continuously to allow quantification of variability and complexity. Muscle activity was measured from the vastus lateralis electromyogram (EMG), and MVCs were used to quantify force generating capacity.

### Dynamometry

During all visits, participants sat in the chair of a Biodex System 4 isokinetic dynamometer (Biodex Medical Systems Inc., New York, USA), initialised and calibrated according to the manufacturer’s instructions. The participant’s right leg was attached to the lever arm of the dynamometer, with the seating position adjusted to ensure that the lateral epicondyle of the femur was in line with the axis of rotation of the lever arm. Participants sat with relative hip and knee angles of 85° and 90°, respectively, with full extension being 0°. The lower leg was securely attached to the lever arm above the malleoli with a padded Velcro strap, while straps secured firmly across the thigh, waist and shoulders prevented any extraneous movement and the use of the hip extensors during the isometric contractions. The seating position was recorded during the familiarisation and replicated during subsequent visits.

### Surface EMG

The EMG of the vastus lateralis was sampled using Ag/AgCl electrodes (36 x 40 mm; Ambu WhiteSensor 3351-S/RT; Ambu A/S, Copenhagen, Denmark). Prior to attachment of the electrodes, the skin of the participants was shaved, abraded, and cleaned with an alcohol swab to reduce impedance. Electrodes were placed on prepared skin over the mid-belly of the muscle (halfway between the anterior superior iliac spine and patella), parallel to the approximate alignment of the muscle fibres. A reference electrode was placed on prepared skin medial to the tibial tuberosity. The EMG signals were sampled using Ultium EMG (Noraxon USA Inc., Scotsdale, Arizona, USA).

### Protocol

**Fig 1:**
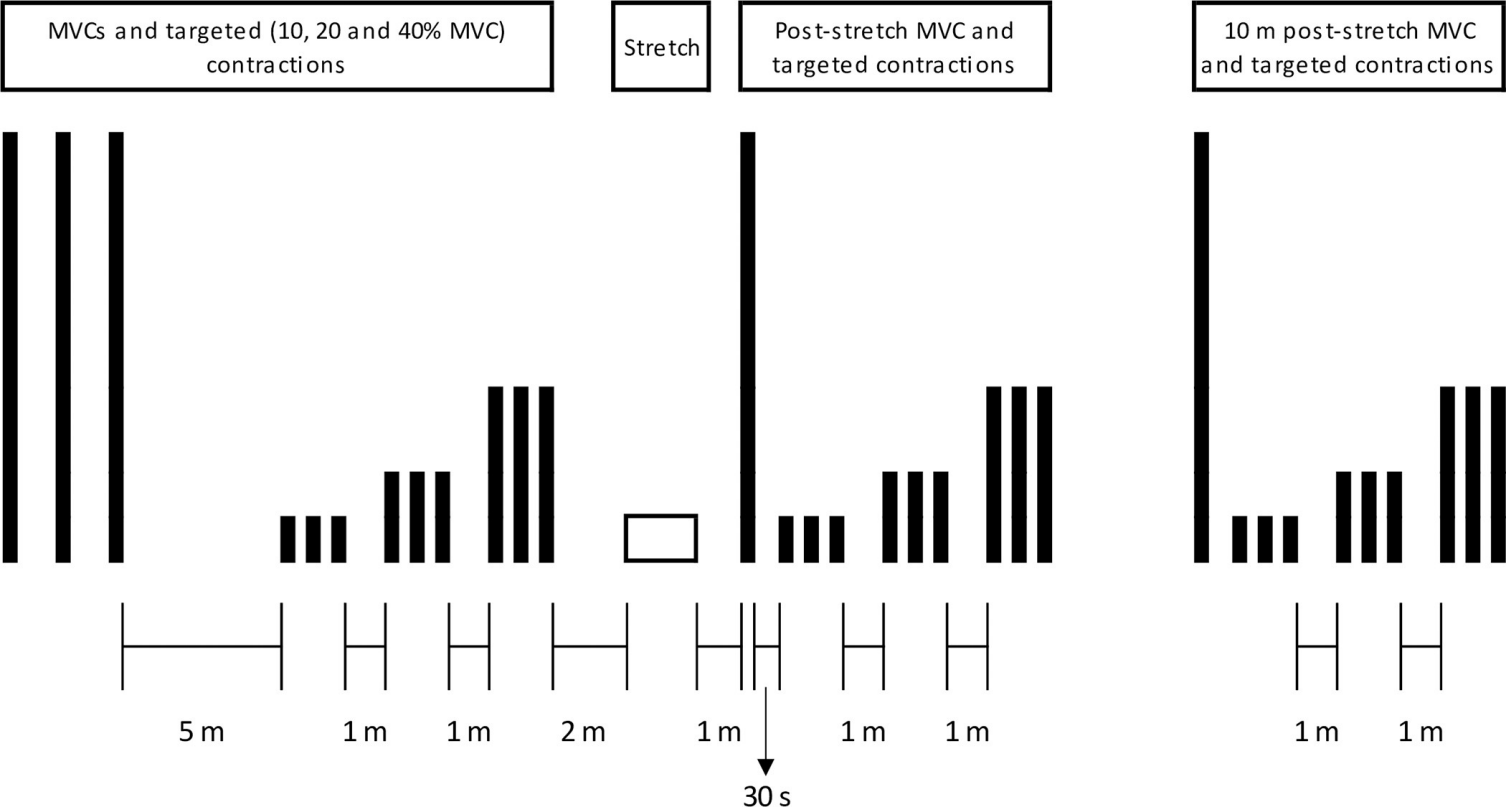
Graphical representation of the experimental protocol.

All visits began with the instrumentation of the participants and the (re-)establishment of the correct dynamometer seating position. Participants performed two tasks: 1) MVCs, to assess muscle force generating capacity; and 2) constant force task (i.e., targeted isometric contractions), to assess muscle force control. These measures were taken prior to, one minute after completion of the SS conditions (to allow re-seating of participant in the dynamometer) and ten minutes after completion of the SS protocol. The protocol is summarised in Fig 1.

### Maximal voluntary contraction task

Prior to SS, participants performed a series of brief (3-second) isometric knee extension MVCs. These MVCs were separated by sixty seconds of rest and continued until the peak force in three consecutive contractions were within 5% of each other. In the majority of cases, participants achieved values within 5% of each other in the first three contractions performed. In no cases, did it take more than four contractions to achieve three consecutive contractions within 5% of each other. Participants were given a 3-second countdown, followed by strong, consistent verbal encouragement to maximise force. The first MVC was used to establish the fresh maximal EMG signal, against which subsequent EMG signals were normalised (*Data analysis*; see below). In the measures taken immediately and ten minutes after the SS protocol, only one MVC was performed at each time point.

### Constant force task

After the establishment of maximal force, participants rested for five minutes before performing a series of intermittent isometric knee extensor contractions at 25, 50 and 75% MVC, in order to establish force control across the spectrum of voluntary forces. The target forces were determined from the highest instantaneous force obtained during the preceding MVCs. Participants performed three contractions at each intensity, with contractions held for six seconds and separated by four seconds of rest [28]. The intensities were performed in a randomised order, with sixty seconds rest separating them. Participants were instructed to match their instantaneous force with a target bar superimposed on a display in front of them and were required to continue matching this for as much of the six second contraction as possible.

### Static stretching intervention

After performing the pre-stretching MVC and constant force tasks, participants rested for two minutes before performing one of four SS conditions: 1) no stretch (control); 2) 30-second stretch; 3) 60-second stretch; and 4) 120-second stretch. The stretch performed was a standing quadriceps stretch, held at the position of onset of discomfort [5]. Participants stood upright with their right knee flexed and heel pulled towards their buttocks, while extending the hip [11]. Participants held the stretch continuously for the full 30, 60 or 120 seconds. In the no stretch condition, participants walked around the laboratory for 120 seconds with this activity equivalent to the longest stretch duration. The purpose of this was to maintain muscle temperature at a similar level to that achieved by the SS.

Immediately after the cessation of stretching, participants were re-seated in the dynamometer. The post-stretch MVC task, consisting of only a single MVC, commenced one minute after the cessation of stretching. The post-stretch force control task commenced a further thirty seconds later and consisted of the same procedure, and target forces, used prior to the SS intervention. This procedure was repeated ten minutes after the cessation of stretching.

### Data acquisition and participant interface

The isokinetic dynamometer was connected via a custom-built cable to a CED Micro 1401–4 (Cambridge Electronic Design, Cambridge, UK). Data were sampled at 1 kHz and collected in Spike2 (Version 10; Cambridge Electronic Design, Cambridge, UK). The EMG signals were sampled at 2 kHz, band-pass filtered (10–500 Hz) and collected in MR3 software (Noraxon USA Inc., Scotsdale, Arizona, USA). The force and EMG signals were synchronised by means of a custom-built trigger cable.

A chart containing the instantaneous force was projected onto a screen placed ~1 m in front of the participant. A scale consisting of a thin line (1 mm thick) was superimposed on the force chart and acted as a target, so that participants were able to match their instantaneous force output to the target during each contraction.

### Data analysis

#### Muscle force

Muscle force data was analysed using code written in MATLAB R2017a (The MathWorks, Massachusetts, USA). The mean and peak force for each contraction were determined. Measures of muscle force control were calculated based on the steadiest five seconds of each contraction, identified by MATLAB as the five seconds containing the lowest SD [21, 22, 28].

The magnitude of variability in the force output of each contraction was measured using the SD, which provides a measure of the absolute magnitude of variability in a time-series, and the CV, which provides a measure of the magnitude of variability in a time-series normalised to the mean of the time-series [29].

The complexity of force output was examined using multiple time domain analyses, as recommended by Goldberger *et al*. [20]. The regularity of force output was determined using ApEn [18] and the temporal fractal scaling of force was estimated using the DFA α scaling exponent [19]. The calculations of ApEn and DFA are detailed in Pethick *et al*. [21]. In brief, ApEn was calculated with the template length, *m*, set at 2 and the tolerance, *r*, set at 10% of the SD of force output, as per the recommendations of Forrest *et al*. [30] and as used in numerous previous studies investigating muscle force complexity in neuromuscular fatigue [22, 28, 31]. DFA was calculated across time scales (57 boxes ranging from 1250 to 4 data points). Sample entropy was also calculated, though as demonstrated by Pethick *et al*. [21], this measure does not differ from ApEn when 5000 data points are used in its calculation (i.e., 5 seconds of contraction with lowest standard deviation multiplied by 1 kHz sampling frequency), as was the case in the present study. The ApEn and DFA measurements taken in the present study both demonstrate very good reliability, as indicated by intra-class correlation coefficients of 0.944 and 0.892, respectively.

#### EMG

The EMG outputs from the vastus lateralis for each contraction were band-pass filtered (10–500 Hz) and full-wave rectified. The average rectified EMG (arEMG) was calculated and normalised by expressing the arEMG as a fraction of the arEMG obtained during a three second MVC from the fresh muscle performed at the beginning of each visit. Technical issues meant that EMG data was only available for 9 of the 12 participants.

#### Statistics

Data were analysed in SPSS (version 28; IBM Corporation, USA). All data are presented as means ± SD. Data were tested for normality using the Shapiro-Wilk test. A two-way ANOVA with repeated measures and two intra-subject factors (four SS conditions and three time points) was used to determine significant differences in MVC. Similar two-way ANOVAs with repeated measures and two intra-subject factors (condition and time points) were also used to determine significant differences in force control measures (SD, CV, ApEn and DFA α) and arEMG for each of the three submaximal contraction intensities (25, 50 and 75% MVC) that constituted the constant force task. The principal purpose of this analysis was, however, to assess the effects of each of the SS conditions separately, rather than compare across them. When main effects were observed, Bonferroni-adjusted 95% paired-samples confidence intervals were used to identify specific differences. Cohen’s *d* was calculated for pre- vs. post-SS and pre- vs. 10 minutes post-SS values for each variable, with effect sizes interpreted as trivial <0.2, small 0.2 ≤ d < 0.5, medium 0.5 ≤ d < 0.8, and large ≥ 0.8. The magnitude of change in each variable was calculated and correlations between this and stretch duration were analysed using Pearson’s product moment correlation (*r*). Results were deemed statistically significant when *P* < 0.05.

## Results

### MVC

There was a significant condition x time interaction for MVC (*F* = 6.306, *P* < 0.001). There was no significant change in MVC from pre- to post-SS in the no-stretch (control) condition (289.1 ± 45.9 to 287.3 ± 46.8 N·m; 95% paired-samples confidence intervals [CIs]: –3.6, 7.1 N·m; *d* = 0.04). Significant decreases in MVC were, however, evident in the 30-second (297.5 ± 53.4 to 283.8 ± 49.3 N·m; CIs: –22.0, –5.5 N·m; *d* = 0.27), 60-second (300.0 ± 55.1 to 276.2 ± 51.7 N·m; CIs: –35.9, –11.6 N·m; *d* = 0.45) and 120-second (308.2 ± 49.4 to 278.6 ± 46.0 N·m; CIs: –46.4, –12.8 N·m; *d* = 0.62) stretch conditions. Moreover, these significant decreases were still evident 10 minutes after the stretch in the 60-second (277.3 ± 58.9 N·m; CIs: –40.6, –4.7 N·m; *d* = 0.40) and 120 second (280.9 ± 55.3 N·m; CIs: –49.1, –5.6 N·m; *d* = 0.52) stretch conditions. A significant correlation was observed between the magnitude of decrease in MVC (no-stretch = 0.7 ± 2.5%; 30-second = 4.8 ± 3.4%; 60-second = 8.8 ± 5.3%; 120-second = 11.0 ± 8.2%) and SS duration (*r* = –0.58, *P* < 0.001).

### Variability and complexity

**Fig 2:**
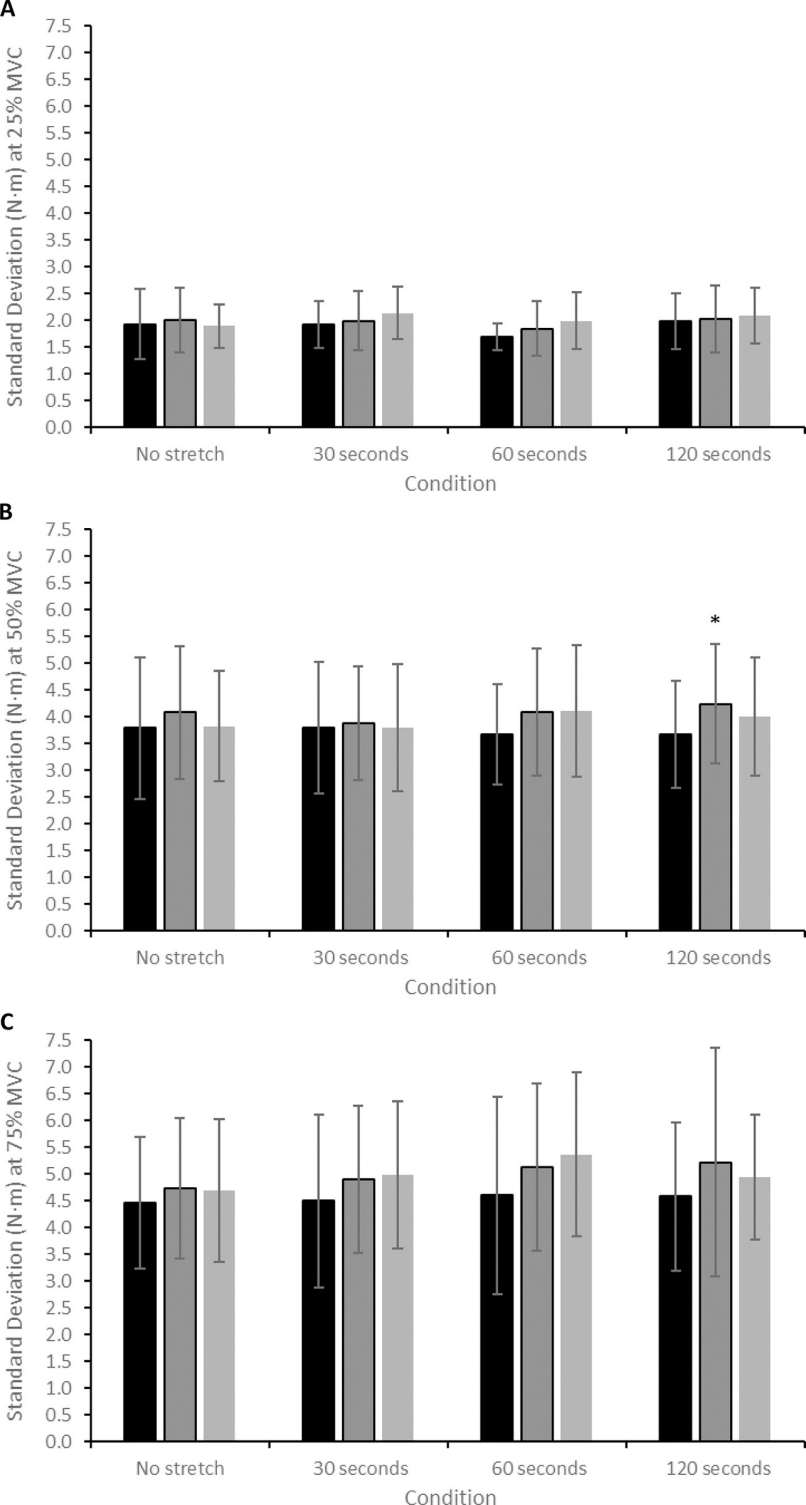
(A) Change in SD during contractions at 25% MVC after each of the static stretch conditions. (B) Change in SD during contractions at 50% MVC after each of the static stretch conditions. (C) Change in SD during contractions at 75% MVC after each of the static stretch conditions. * = significant difference (*P* < 0.05) compared to the pre-stretch value.

There was no significant interaction effect for SD during contractions performed at 25% MVC (*F* = 1.084, *P* = 0.381). There was a significant effect of time for SD during contractions at 50% MVC (*F* = 3.615, *P* = 0.044), with post-hoc testing revealing that SD at 50% MVC significantly increased from pre- to post-SS in the 120-second stretch condition (3.68 ± 1.00 to 4.24 ± 1.11 N·m; CIs: 0.09, 1.0 N·m; *d* = 0.53; Fig 2B; Table 1). There was a significant effect of time for SD during contractions at 75% MVC (*F* = 3.788, *P* = 0.039), though post-hoc testing revealed no specific differences. There was no significant correlation between the magnitude of change in SD across any contraction intensity and SS duration (all *P >* 0.05; Table 2).

**Table 1:** Effect sizes (vs. pre values) for force control and EMG measures at each contraction intensity in each of the stretch conditions.

	No stretch (control)	30-second stretch	60-second stretch	120-second stretch
SD				
Post-25% MVC (N·m)	0.11	0.14	0.35	0.07
10 mins post-25% (N·m)	0.07	0.46	0.70	0.21
Post-50% MVC (N·m)	0.23	0.08	0.40	0.53
10 mins post-50% (N·m)	0.03	0.01	0.40	0.30
Post-75% MVC (N·m)	0.21	0.27	0.31	0.36
10 mins post-75% (N·m)	0.18	0.32	0.44	0.28
CV				
Post-25% MVC (%)	0.11	0.19	0.35	0.08
10 mins post-25% (%)	0.17	0.58	0.86	0.30
Post-50% MVC (%)	0.34	0.22	0.51	0.49
10 mins post-50% (%)	0.23	0.14	0.62	0.31
Post-75% MVC (%)	0.35	0.53	0.37	0.57
10 mins post-75% (%)	0.24	0.51	0.55	0.42
ApEn				
Post-25% MVC	0.20	0.00	0.70	0.00
10 mins post-25%	0.10	0.49	1.23	0.33
Post-50% MVC	0.17	0.10	0.55	0.44
10 mins post-50%	0.09	0.00	0.55	0.33
Post-75% MVC	0.33	0.43	0.40	0.78
10 mins post-75%	0.22	0.42	0.73	0.59
DFA				
Post-25% MVC (α)	0.23	0.14	0.18	0.22
10 mins post-25% (α)	0.40	0.14	0.54	0.50
Post-50% MVC (α)	0.25	0.00	0.66	0.35
10 mins post-50% (α)	0.00	0.00	0.66	0.38
Post-75% MVC (α)	0.27	0.12	0.00	0.38
10 mins post-75% (α)	0.27	0.37	0.40	0.13

MVC = maximal voluntary contraction; SD = standard deviation; CV = coefficient of variation; ApEn = approximate entropy; DFA = detrended fluctuation analysis; arEMG = average rectified EMG.

**Table 2:** Magnitude of change in force control and EMG measures at each contraction intensity in each of the stretch conditions.

	No stretch (control)	30-second stretch	60-second stretch	120-second stretch
SD				
25% MVC Δ pre to post (N·m)	0.07 ± 0.38	0.07 ± 0.51	0.14 ± 0.38	0.04 ± 0.40
50% MVC Δ pre to post (N·m)	0.29 ± 1.00	0.09 ± 0.72	0.42 ± 0.65	0.55 ± 0.58
75% MVC Δ pre to post (N·m)	0.27 ± 0.63	0.39 ± 0.75	0.53 ± 1.16	0.64 ± 1.05
CV				
25% MVC Δ pre to post (%)	0.08 ± 0.62	0.10 ± 0.73	0.16 ± 0.43	0.06 ± 0.51
50% MVC Δ pre to post (%)	0.22 ± 0.48	0.11 ± 0.46	0.26 ± 0.40	0.40 ± 0.44
75% MVC Δ pre to post (%)	0.19 ± 0.49	0.22 ± 0.33	0.37 ± 0.49	0.49 ± 0.54
ApEn				
25% MVC Δ pre to post	0.02 ± 0.08	0.01± 0.06	0.02± 0.06	0.06 ± 0.06
50% MVC Δ pre to post	0.02 ± 0.07	0.01± 0.08	0.05 ± 0.06	0.05 ± 0.05
75% MVC Δ pre to post	0.03 ± 0.07	0.04 ± 0.08	0.04 ± 0.08	0.06 ± 0.04
DFA α				
25% MVC Δ pre to post (α)	0.03± 0.06	0.01± 0.05	0.01± 0.04	0.04± 0.06
50% MVC Δ pre to post (α)	0.02± 0.05	0.00 ± 0.00	0.03± 0.05	0.02± 0.04
75% MVC Δ pre to post (α)	0.02 ± 0.07	0.01 ± 0.06	0.00 ± 0.03	0.03 ± 0.01
arEMG				
25% MVC Δ pre to post (%MVC)	0.5 ± 5.2	2.0 ± 6.8	5.4 ± 7.8	2.2 ± 8.6
50% MVC Δ pre to post (%MVC)	2.2 ± 8.2	2.5 ± 7.4	5.7 ± 9.3	7.8 ± 3.1
75% MVC Δ pre to post (%MVC)	1.5 ± 2.3	4.1 ± 9.2	5.9 ± 4.9	6.3 ± 5.6

MVC = maximal voluntary contraction; SD = standard deviation; CV = coefficient of variation; ApEn = approximate entropy; DFA = detrended fluctuation analysis; arEMG = average rectified EMG.

**Fig 3:**
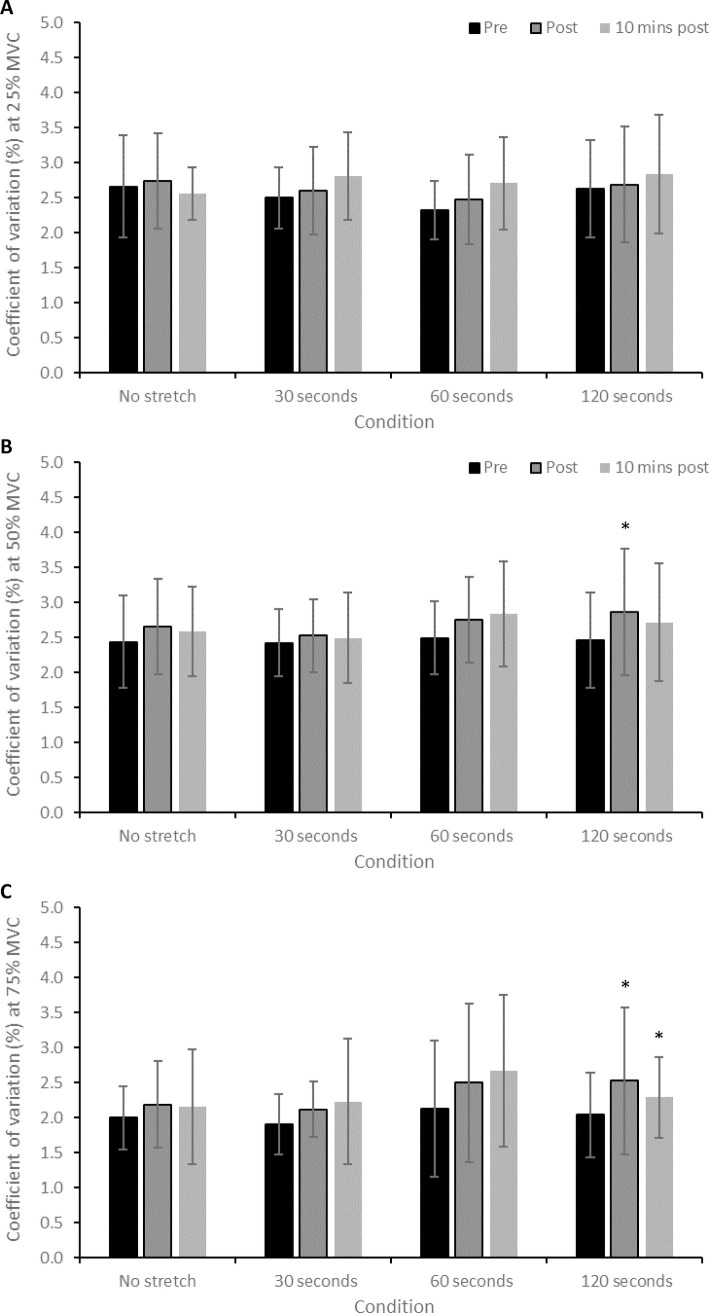
(A) Change in CV during contractions at 25% MVC after each of the static stretch conditions. (B) Change in CV during contractions at 50% MVC after each of the static stretch conditions. (C) Change in CV during contractions at 75% MVC after each of the static stretch conditions. * = significant difference (*P* < 0.05) compared to the pre-stretch value.

There was no significant interaction effect for CV during contractions performed at 25% MVC (*F* = 1.445, *P* = 0.211). There was a significant effect of time for contractions at 50% MVC (*F* = 4.981, *P* = 0.016), with post-hoc testing revealing that CV at 50% MVC significantly increased from pre- to post-SS in the 120-second stretch condition (2.47 ± 0.68 to 2.86 ± 0.90%; CIs: 0.04, 0.07%; *d* = 0.49; Fig 3B; Table 1). There was a significant effect of time for CV during contractions at 75% MVC (*F* = 5.913, *P* = 0.009), with post-hoc testing revealing that CV at 75% MVC significantly increased from pre- to post-SS in the 120-second stretch condition (2.04 ± 0.60 to 2.53 ± 1.05%; CIs: 0.04, 0.9%; *d* = 0.57; Fig 3C; Table 1). Furthermore, this increase at CV at 75% MVC was still evident 10 minutes after the stretch (2.04 ± 0.60 to 2.29 ± 0.58; CIs: 0.02, 0.5%; *d* = 0.42; Fig 3C; Table 1). There was no significant correlation between the magnitude of change in CV across any contraction intensity and SS duration (all *P >* 0.05; Table 2).

**Fig 4:**
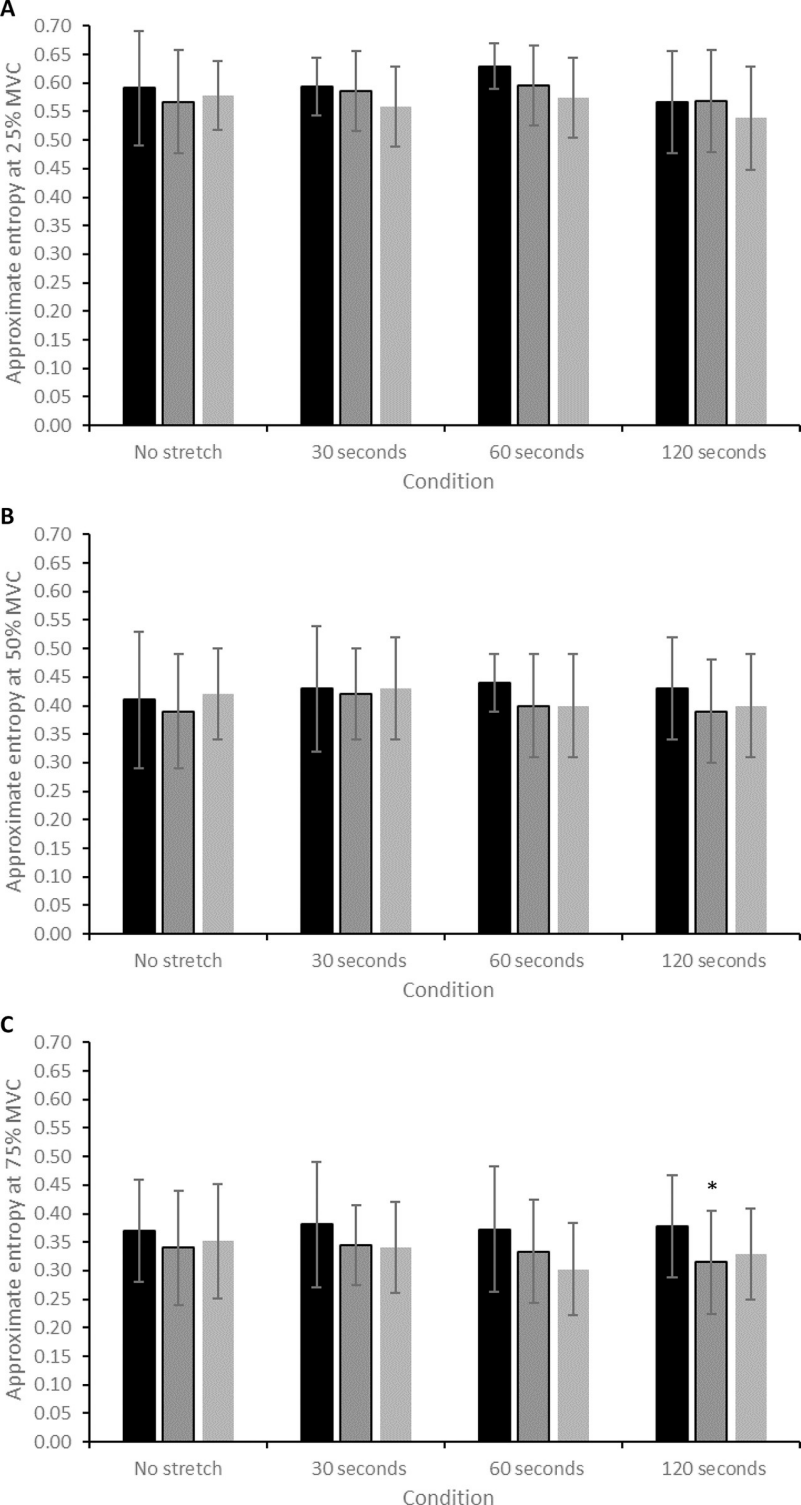
(A) Change in ApEn during contractions at 25% MVC after each of the static stretch conditions. (B) Change in ApEn during contractions at 50% MVC after each of the static stretch conditions. (C) Change in ApEn during contractions at 75% MVC after each of the static stretch conditions. * = significant difference (*P* < 0.05) compared to the pre-stretch value.

There was a significant effect of time on ApEn during contractions performed at 25% MVC (*F* = 6.504, *P* = 0.006), though post-hoc testing revealed no specific differences. There was no significant interaction effect for ApEn on contractions at 50% MVC (*F* = 0.811, *P* = 0.565). There was a significant effect of time for ApEn on contractions at 75% MVC (*F* = 5.657, *P* = 0.010), with post-hoc testing revealing that ApEn at 75% MVC significantly decreased from pre- to post-SS in the 120-second stretch condition (0.38 ± 0.09 to 0.31 ± 0.09; CIs: –0.09, –0.03; *d* = 0.78; Fig 4C; Table 1). There was no significant correlation between the magnitude of change in ApEn across any contraction intensity and SS duration (all *P >* 0.05; Table 2).

**Fig 5:**
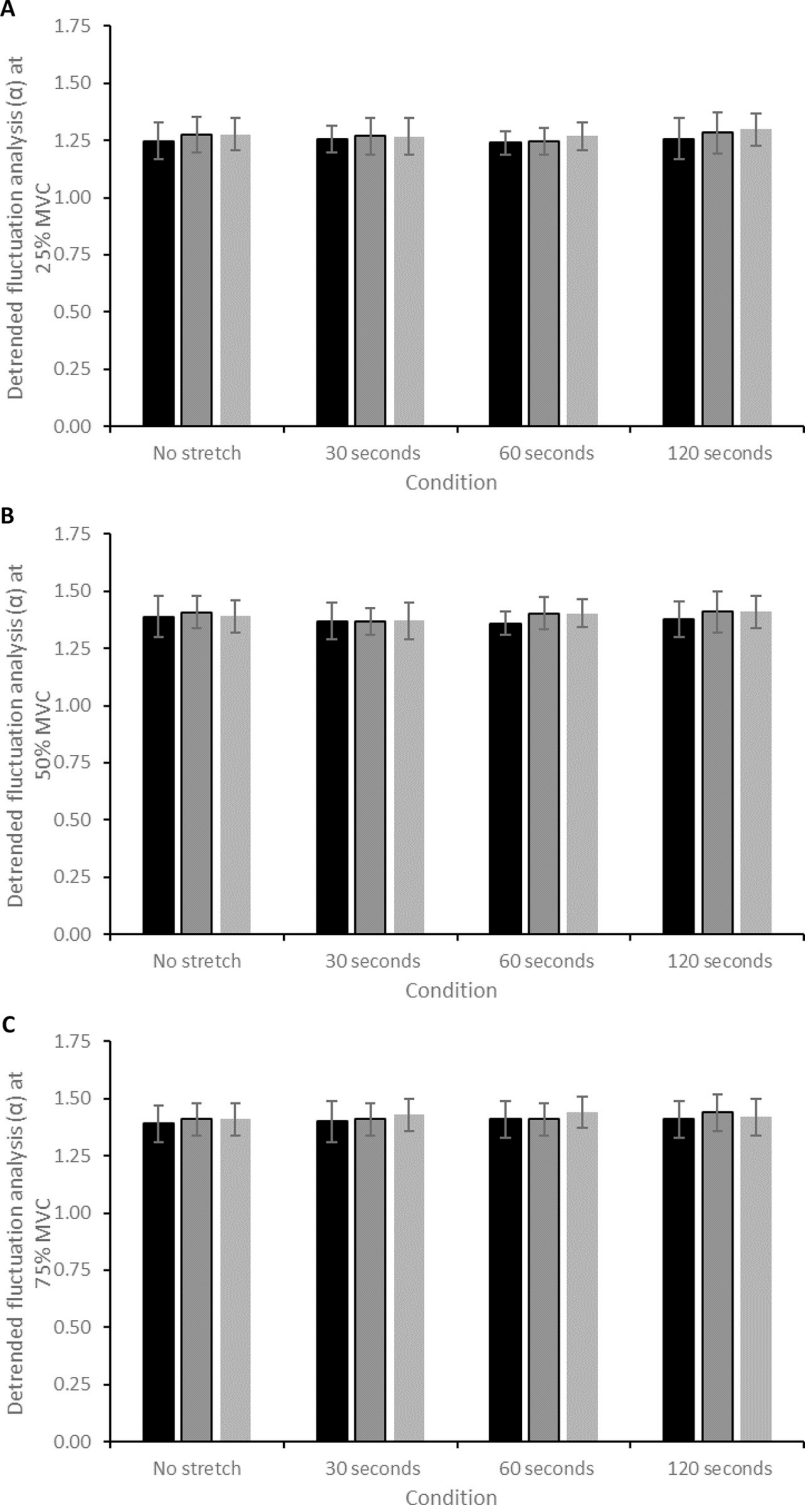
(A) Change in DFA α during contractions at 25% MVC after each of the static stretch conditions. (B) Change in DFA α during contractions at 50% MVC after each of the static stretch conditions. (C) Change in DFA α during contractions at 75% MVC after each of the static stretch conditions.

There was a significant effect of time on DFA α during contractions performed at 25% MVC (*F* = 6.049, *P* = 0.008), though post-hoc testing revealed no specific differences. There was no significant interaction effect on DFA α during contractions at 50% MVC (*F* = 3.426, *P* = 0.051), nor was there a significant interaction effect during contractions at 75% MVC (*F* = 0.496, *P* = 0.809; Fig 5; Table 1). There was no significant correlation between the magnitude of change in DFA α across any contraction intensity and SS duration (all *P >* 0.05; Table 2).

### EMG

**Fig 6:**
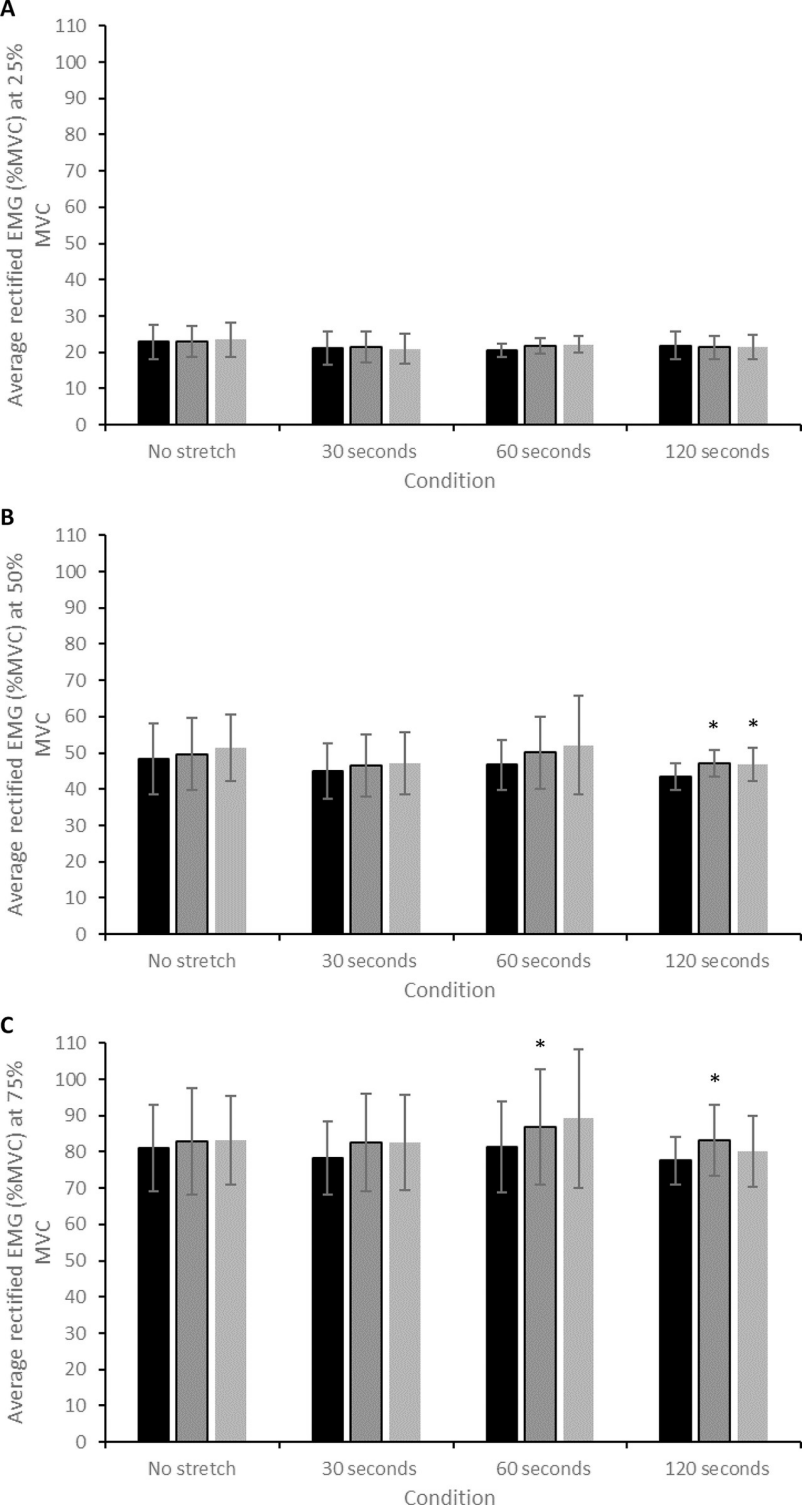
(A) Change in arEMG during contractions at 25% MVC after each of the static stretch conditions. (B) Change in arEMG during contractions at 50% MVC after each of the static stretch conditions. (C) Change in arEMG during contractions at 75% MVC after each of the static stretch conditions. * = significant difference (*P* < 0.05) compared to the pre-stretch value.

There was a significant interaction effect for arEMG during contractions performed at 25% MVC (*F* = 2.937, *P* = 0.016), though post-hoc testing revealed no specific differences. There was a significant effect of time for arEMG during contractions at 50% MVC (*F* = 6.958, *P* = 0.007), with post-hoc testing revealing that arEMG at 50% significantly increased from pre- to post-SS in the 120-second stretch condition (43.5 ± 3.6 to 47.2 ± 3.7%; CIs: 2.1, 5.2%; *d* = 1.01; Fig 6B; Table 1). Furthermore, this increase in arEMG at 50% MVC was still evident 10 minutes after the stretch (43.5 ± 3.6 to 46.8 ± 4.7; CIs: 0.04, 6.7%; *d* = 0.79; Fig 6B; Table 1). There was a significant effect of time on arEMG during contractions at 75% MVC (*F* = 8.522, *P* = 0.003), with post-hoc testing revealing that arEMG significantly increased from pre-to post-SS in the 60-second condition (81.3 ± 12.6 to 86.9 ± 15.9; CIs: 0.2, 10.9%; *d* = 0.39; Fig 6C) and the 120-second condition (77.6 ± 6.5 to 83.2 ± 9.7%; CIs: 0.8, 10.4%; *d* = 0.69; Fig 6C; Table 1). There was no significant correlation between the magnitude of change in arEMG across any contraction intensity and SS duration (all *P* > 0.05; Table 2).

## Discussion

The major novel finding of the present study was that acute SS decreased knee extensor force control during subsequent submaximal isometric contractions. Specifically, 120 seconds of SS increased the magnitude and decreased the complexity of force fluctuations during contractions at 50% and 75% MVC. These responses were accompanied by increased arEMG, suggesting greater activation of the motor unit pool. In contrast, no change in force control was evident during contractions at 25% MVC, nor did stretch durations of ≤60 seconds have an effect on force control. These results indicate that the detrimental effects of prolonged SS extend beyond tasks necessitating maximal force generation to tasks involving generation of task-relevant and precise levels of force during moderate- to high-intensity submaximal contractions.

### Effect of static stretching on force control

The present study is the first to demonstrate that prolonged SS can impair knee extensor force control during moderate- to high-intensity contractions. Following the 120-second stretch, the magnitude of force fluctuations significantly increased (greater SD at 50% MVC and greater CV at 50 and 75% MVC; Figs 2 and 3; Table 1), indicative of decreased (i.e., poorer) force steadiness [15, 29]. Moreover, greater CV at 75% MVC was still evident ten minutes after the 120-second stretch, suggesting a longer-lasting effect of prolonged SS on force control, similar to that observed for maximal force generating capacity [32]. In contrast, neuromuscular fatigue-induced increases in knee extensor force CV are reversed back to baseline levels within 10 minutes of the cessation of exercise [31]. The 120-second stretch also significantly decreased the complexity of knee extensor force fluctuations (decreased ApEn at 75% MVC; Fig 4; Table 1), indicative of increased signal regularity. This change suggests a decreased adaptive capacity of the neuromuscular system [15, 29].

The decrease in muscle force control following prolonged SS could have implications for subsequent exercise performance. It has been demonstrated that variance in endurance time is associated with baseline CV [33], such that a better ability to control force is associated with greater fatigue resistance. Consequently, the presently observed SS-induced decrease in force control could result in taking less time to reach limiting values during an endurance task [34]. Similarly, lower knee extensor force CV and greater sample entropy during contractions at 40% MVC are associated with greater dynamic balance (anterior reach during Y balance test; [35]), a parameter of vital importance for most sports. The presently observed decrease in force control could, therefore, contribute to an increased risk of falls and subsequent injury. However, whether the observed changes are of practical relevance for subsequent performance requires further investigation. Nevertheless, it may be advisable to wait in excess of ten minutes after performing prolonged SS before engaging in any activity that necessitates force control.

In agreement with Kay and Blazevich [12], a significant correlation was observed between SS duration and the decrease in MVC, indicating a dose-response relationship. Despite this, knee extensor force control was only affected by the 120-second stretch, with SD, CV and ApEn all significantly changing. This suggests that maximal force generating capacity is more readily affected by perturbation than force control; a supposition supported by the observation of decreasing MVC but no change in either magnitude- or complexity-based measures of force control following 30 minutes of contractions performed below the critical torque [22]. Force control was also only affected during contractions at 50 and 75% MVC, likely due to the disproportionate effect the decrease in MVC has on those targets. For example, if MVC was 300 N·m and decreased by 11% (the average decrease arising from the 120-second stretch), the original target of 75% MVC would equate to ~84% of the post-stretch MVC while the original target of 25% MVC would equate to ~28% of the post-stretch MVC. An increase in the magnitude of force fluctuations has, however, previously been observed during plantarflexion contractions at 20% MVC, though this was following an accumulated 300 seconds of passive (isokinetic dynamometer-induced) SS [36].

### Physiological basis for change in force control with static stretching

There is considerable evidence implicating reduced neural drive as the mechanism for the SS-induced reduction in maximal force generating capacity [8]. Consequently, an increase in neural drive would be necessary in order achieve the same absolute submaximal force. Following the 120 second stretch, an increase in arEMG at 50 and 75% MVC was observed (Fig 6; Table 1), indicating the need for greater motor unit recruitment and/or firing rate. This is in accord with previous research demonstrating a decrease in motor unit recruitment threshold, an increase in motor unit firing rate and an increase in neural drive following repeated SS totalling 120 seconds [26]. In contrast to the present observations, these findings were only evident during low (10% MVC) but not moderate (35% MVC) intensity contractions.

Muscle force CV is highly coherent with the cumulative motor unit spike train [37]. This suggests that differences in CV between conditions indicate variance in common modulation of motor unit discharge times [16]. Thus, the presently observed increase in CV at 50 and 75% MVC following the 120-second stretch is indicative of greater neural drive and common modulation of motor unit discharge times. It has been speculated that such motor unit behaviour is also likely the cause of decreased force complexity [22]. The exact mechanism(s) for this increase in common modulation of motor unit discharge times following prolonged SS has yet to be elucidated.

It has been suggested that reduced motoneuron excitability, caused by reduced persistent inward current strength, may be responsible for the SS-induced force loss [38]. Persistent inward currents amplify and prolong synaptic input and, therefore, play an important role in facilitating motor unit recruitment and firing rates [39]. This may also offer a plausible, though not the only, hypothesis for the SS-induced reduction in force control. SS may affect the muscle force-length relationship, such that the muscle is operating at shorter fascicle lengths [10]. SS-induced reductions in fascicle length have been demonstrated to be accompanied by elevated EMG activity [36]. Consistent with this, shorter muscle lengths have been demonstrated to be accompanied by greater motor unit discharge times [40] and a greater magnitude and lower complexity of force fluctuations [28].

### Limitations

It could be argued that the present study lacks ecological validity as the 120-second stretch duration may be considered excessive, as this timeframe is not typically utilised in the field by practitioners. This was, however, a deliberate design choice to allow comparison with previous work, much of which has used similarly long stretch durations [2], to investigate the potential of a dose-response relationship between SS duration and changes in force control, and because changes in motor unit behaviour thought to underlie force control are unaffected by SS durations of <60 seconds [2]. Secondly, the design of the study did not include an aerobic component prior to the stretch or any dynamic movements after the stretch. Again, this was a deliberate design choice in order to simply investigate the effect of static stretching alone on force control. A number of recent studies have demonstrated that when static stretching is utilising within a comprehensive warm-up, the effects on subsequent performance are trivial or even positive [41, 42]. Indeed, a recent study investigating the complexity of hamstring EMG output found an increase in sample entropy during isometric contractions at 40% MVC after an aerobic warm-up followed by six sets of 30-second static stretches [43]. This is particularly interesting, as an increase in sample entropy indicates an increase in complexity and is in contrast to the decrease in muscle force complexity observed in the present study. Investigating the effect on force control of static stretching when part of a comprehensive warm-up represents the next logical step for this line of research. The research of Babault *et al*. [43] also highlights that future studies should measure both muscle force and EMG complexity, particularly that derived from the high-density EMG and the cumulative motor unit spike train [44], to further elucidate both the performance implications and mechanistic basis of the SS-induced loss of force control.

## Conclusion

The present study has demonstrated that prolonged SS impairs muscle force control. Specifically, both magnitude- and complexity-based measures of knee extensor force control (indicative of force steadiness and adaptability, respectively) were impaired during moderate- to high-intensity contractions following a 120-second SS. The increase in muscle force CV provides indirect evidence that greater neural input to the muscle was necessary to meet the target force following SS. This was confirmed by the accompanying increase in arEMG, indicating greater activation of the motor unit pool. These results provide further evidence that prolonged SS in isolation (without additional dynamic warm-up activities) impairs muscular performance and that it does so via neural mechanisms.
